# Machine-Learning-Algorithm-Assisted Portable Miniaturized NIR Spectrometer for Rapid Evaluation of Wheat Flour Processing Applicability

**DOI:** 10.3390/foods14101799

**Published:** 2025-05-19

**Authors:** Yuling Wang, Chen Zhang, Xinhua Li, Longzhu Xing, Mengchao Lv, Hongju He, Leiqing Pan, Xingqi Ou

**Affiliations:** 1 School of Agriculture, Henan Institute of Science and Technology, Xinxiang 453003, China; wangyuling634@hist.edu.cn (Y.W.); lixinhua00530@163.com (X.L.); 2 School of Information Engineering, Xinxiang Institute of Engineering, Xinxiang 453700, China; zhangchen.hist@gmail.com; 3 School of Food Science, Henan Institute of Science and Technology, Xinxiang 453003, China; longzhu_xing@163.com (L.X.); mengchao_lv@163.com (M.L.); 4 College of Food Science and Technology, Nanjing Agricultural University, Nanjing 210095, China; pan_leiqing@njau.edu.cn

**Keywords:** machine learning, wheat flour, NIR, sedimentation value, falling number

## Abstract

In this investigation, we established an intelligent computational framework comprising a novel starfish-optimization-algorithm-optimized support vector regression (SOA-SVR) model and a multi-algorithm joint strategy to evaluate the processing applicability of wheat flour in terms of sedimentation value (SV) and falling number (FN) using near-infrared (NIR) data (900–1700 nm) obtained using a miniaturized NIR spectrometer. By employing an improved whale optimization algorithm (iWOA) coupled with a successive projections algorithm (SPA), we selected the 20 most informative wavelengths (MIWs) from the full range spectra, allowing the iWOA/SPA-SOA-SVR model to predict SV with correlation coefficient and root-mean-square errors in prediction (R_P_ and RMSE_P_) of 0.9605 and 0.2681 mL. Additionally, RFE, in combination with the iWOA, identified 30 MIWs and enabled the RFE/iWOA-SOA-SVR model to predict the FN with an R_P_ and RMSE_P_ of 0.9224 and 0.3615 s. The robustness and reliability of the two SOA-SVR models were further validated using 50 independent samples per index, a statistical two-sample F-test, and a *t*-test. In conclusion, the combination of a portable miniaturized NIR spectrometer and an SOA-driven SVR algorithm demonstrated technical feasibility in quantifying the SV and FN of wheat flour, thus providing a novel strategy for the on-site assessment of wheat flour processing applicability.

## 1. Introduction

According to the latest data from the Food and Agriculture Organization of the United Nations (FAO), the global wheat production yield reached approximately 800 million tons in 2023 [1], with the majority allocated to milling purposes [2]. From the advent of fermented bread in ancient Egypt to the formation of current modern industrialized food systems, wheat flour has served as a cornerstone of human food civilization, playing dual roles in both energy supply and cultural continuity [3]. As a primary source of global energy, wheat flour provides essential macronutrients, such as carbohydrates, protein, and fat, as well as micronutrients, including dietary fiber, vitamin B, and minerals. Additionally, wheat flour also contains a variety of bioactive components like phenolic compounds and enzymes that contribute to human health and dietary diversification [4]. As a versatile ingredient, wheat flour is essential to the production of diverse food products ranging from noodles, bread, and pasta to cookies, cakes, and snacks [5]. With the increasing demand for flour-based foods with diverse textures, nutritional profiles, and functional properties, accurate and rapid evaluation of flour processing parameters remains quite important for ensuring product acceptability and market suitability [6].

Sedimentation value (SV), a key indicator of flour processing quality, is usually evaluated using a Zeleny or SDS sedimentation test to quantify the content, functionality, and hydration properties of gluten-forming proteins (glutenin and gliadin), such as water absorption and swelling capacity [7]. High SV values indicate tightly crosslinked gluten networks that lead to superior dough elasticity and air retention, directly correlating with greater bread volume and enhanced noodle texture [8]. Falling number (FN), the other widely recognized important parameter, is used for evaluating α-amylase activity and starch gelatinization characteristics [9]. By quantifying starch degradation kinetics, FN can be used to indirectly assess pre-harvest sprouting damage and processing adaptability [10]. Furthermore, FN is directly related to the rheological properties of dough [11], with a high FN causing delayed fermentation and undesirably dense baked goods, while a low FN leads to excessive gelatinization and structural incompleteness of products like noodles. As two complementary analytical parameters, the SV and FN provide objective and quantifiable data that can be used to replace subjective sensory assessments to enable comprehensive quality control across the whole wheat chain, from varietal selection and agronomic practices to milling optimization and end-product production, thus ensuring precise flour classification and processing efficiency [12,13].

Traditional methods for determining the SV and FN of wheat flour primarily rely on chemical assays, which are time-consuming and destructive and must be performed manually. Although specialized laboratory instruments (e.g., sedimentation centrifuges and automated FN analyzers) have been developed to standardize SV and FN measurements [14], these methods are still hindered by some application challenges, especially in terms of industrial-scale and on-site detection. In addition, these devices are usually oversized and lack portability, making on-site detection (e.g., at farms or storage facilities) technically infeasible. Near-infrared (NIR) spectroscopy has emerged as a powerful analytical tool offering impressive advantages such as rapid analysis, non-destructiveness, no requirement for reagents, and user-friendly protocols [15]. It has been successfully applied to evaluate wheat flour quality, including physicochemical attributes (moisture, protein, ash, and color) [16], gluten content, and rheological properties [17]. NIR spectra always contain overlapping and interference information and must be analyzed using chemometrics or artificial intelligence algorithms. Machine learning can facilitate efficient spectral data processing and predictive modeling through automated extraction of important spectral features from complex datasets, thereby significantly enhancing analytical throughput and prediction accuracy [18]. The integration of NIR spectroscopy with machine learning algorithms not only overcomes the methodological limitations of traditional techniques but also enables rapid in situ quality assessment, providing a feasible solution for industrial quality control. In recent years, NIR combined with machine learning has been successfully applied for the quality evaluation of wheat flour in the detection of chemical components and illegal additives. As shown in Table 1, different machine learning algorithms have demonstrated good performance in mining NIR spectra, further supporting the effectiveness and efficiency of machine learning-assisted NIR modeling.

**Figure 1:**
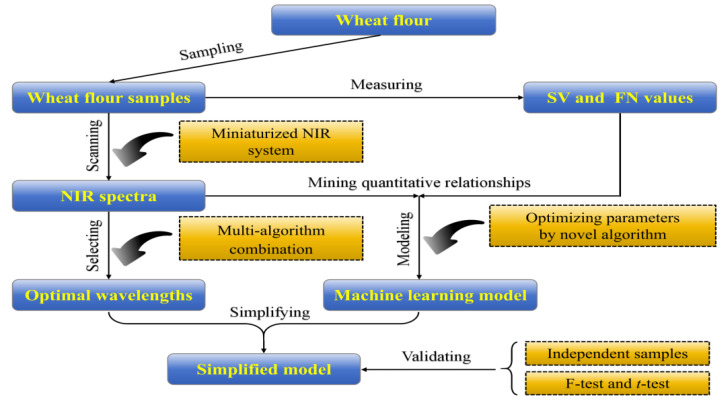
Flowchart depicting the use of a miniaturized NIR system in tandem with a machine learning algorithm for the prediction of the SV and FN of wheat flour.

To extend the applications of NIR in grain science, especially with respect to miniaturized NIR spectrometers, we investigated the potential of a machine learning-assisted portable miniaturized NIR spectroscope for use in the determination of the SV and FN of wheat flour in a convenient, efficient, and non-invasive way. To the best of our knowledge, this study constitutes the first implementation of micro NIR–machine learning integration for the rapid and nondestructive measurement of these key quality indicators, advancing the precise quality characterization of wheat flour. A flowchart illustrating the overall research process is shown in Figure 1.

## 2. Materials and Methods

### 2.1. Flour Preparation

Flour (80 mesh sieve; moisture, 11 ± 0.5%) made from different wheat varieties harvested from different places (Bainong 207 (Xinxiang City), Bainong 307 (Xinxiang City), Bainong 607 (Xinxiang City), Bainong 627 (Xinxiang City), Bainong 627 (Anyang City), Bainong 627 (Hebi), Bainong 627 (Hefei), Bainong 627 (Huai’an), Bainong 627 (Huainan City), Bainong 627 (Lianyungang City), Bainong 627 (Luoyang City), Bainong 627 (Xinxiang City), Bainong 627 (Suzhou), Bainong 627 (Weinan City), Bainong 627 (Xinxiang City), Bainong 627 (Xihua City), Bainong 627 (Xuchang City), Bainong 627 (Zhengzhou City), Bainong 627 (Yangling City), Bainong 697 (Xinxiang City), Xinmai 26 (Xinxiang City), Xinmai 45 (Xinxiang City), Xinmai 58 (Xinxiang City), Zhengmai 366 (Xinxiang City), and Zhoumai 36 (Xinxiang City)) in 2023 and 2024 was supplied by a local milling factory. Approximately 20 duplicate flour batches for each variety from each year were finally prepared, and a total of 921 and 904 flour samples were finally obtained for SV and FN evaluation, respectively.

### 2.2. NIR Calibration and Acquisition

In this study, a pocket-sized NIR spectrometer (NIR-S-G1, InnoSpectra Technology, Hsinchu City, Taiwan) was used. It is composed of an illumination source (2 × 0.7 W, built-in tungsten filament lamps), a sampling module with a sapphire scan window, a digital light-processing-based post-dispersive optical engine, a Bluetooth low-energy module, an operation panel, a rechargeable lithium-ion battery pack, and an electromagnetic compatibility shielding case. The NIR device covers a wavelength range of 900–1700 nm at 2.6 nm intervals (360 wavelengths).

Before the experiment was conducted, a laptop computer equipped with spectral acquisition software (ISC WinForms SDK GUI v3.9, InnoSpectra Technology, Taiwan) was connected to the NIR spectrometer via USB interface. Afterwards, a standard white tile (99.99% reflectance) was placed directly against the sapphire scan window of the NIR spectrometer and scanned to perform NIR calibration. Then, the exposure time and the average number of scans were set to 0.635 ms and 10, respectively. Finally, a flour sample was put on a glass plate (diameter, 6 cm), and the window of the NIR device was pointed at the surface of the flour sample in order to scan it 5 times to acquire an average spectrum representing the sample. By repeating this procedure, we obtained a total of 921 and 904 raw spectra and used them for the further measurement of SV and FN, respectively.

### 2.3. Measurement of SV and FN

After spectral acquisition, the SV (in reference to the sodium dodecyl sulfate SV test) was determined using AACC International Method 56–70. The FN was measured by using an apparatus (FN 1000, Perten Instruments, Huddinge, Sweden) according to AACC International Method 56–81.01. Each index was measured three times and averaged for use. The SV values of the 921 wheat flour samples were measured and determined to range from 17.8 mL to 42.4 mL with standard deviation (SD) of 6.4 mL, while the FN values of the 904 wheat flour samples had a range of 241–450 s with SD of 38 s. Utilizing Scikit-learn tool, 80% and 20% of samples were randomly classified into training and prediction sets, respectively, to quantify SV and FN.

### 2.4. Machine Learning Modeling and Performance Evaluation

In support vector regression (SVR), an extension of the support vector machine (SVM) framework for continuous variable prediction, a regression hyperplane is constructed through the epsilon-insensitive loss function, with the prediction error controlled within the preset epsilon range [24]. SVR is used for solving the convex optimization problem, and it is mathematically expressed as a formula below:

$$\min\frac{1}{2}{\left\|W\right\|}^{2}+C\sum \left(\xi_{i}+\xi_{i}^{*}\right)$$

where $\frac{1}{2}{\left\|W\right\|}^{2}$ is the width of the margin, *C* is a regularization parameter, and *ξ* and ξ^*^ are relaxation variables.

**Figure 2:**
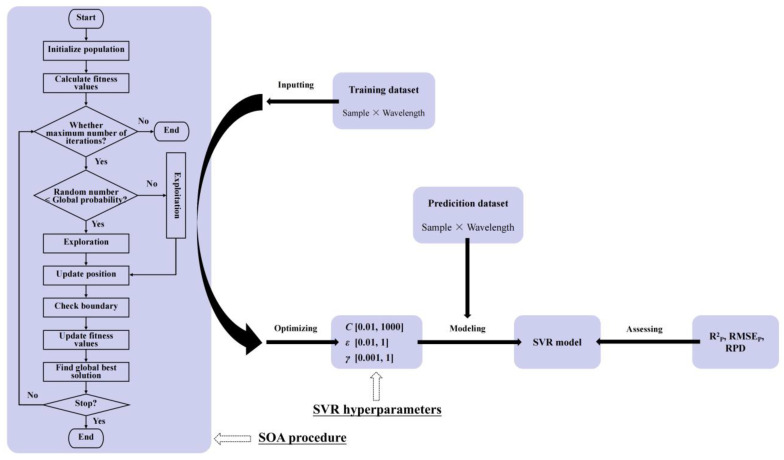
Flowchart depicting the use of SOA to optimize SVR hyperparameters and conduct SVR modeling.

SVR is a supervised learning algorithm and performs well when applied to small quantities of samples and high-dimensional and nonlinear regression tasks. SVR particularly excels at handling complex datasets with nonlinear patterns or high-dimensional features, outperforming traditional regression methods (e.g., linear regression) that often fail to capture intricate relationships [25]. The performance of SVR relies on several critical hyperparameters, including kernel functions (e.g., linear, polynomial, radial basis function (RBF), and sigmoid) for nonlinear fitting by mapping data to high dimensional space, *C* for controlling the trade-off between model complexity and error tolerance, epsilon (*ε*) for defining the width of the error-insensitive tube around the regression line, and kernel-specific parameters (e.g., *γ* for RBF kernels). Proper adjustment of these parameters through grid searches or Bayesian optimization is essential to balance model stability, prediction accuracy, and generalization capability, making SVR particularly valuable in spectral data analysis [26]. Starfish optimization algorithm (SOA), a novel meta-heuristic optimization algorithm based on the crowd behavior of starfish, was proposed by researchers inspired by the foraging, movement, and regeneration patterns of starfish in nature [27]. Through the synergistic interaction of tentacles, starfish explore food resources efficiently and, at the same time, show a strong ability to adapt to the environment, and these characteristics are abstracted into search strategies and optimization mechanisms in this algorithm. The SOA follows a metaheuristic framework structured around two core phases, exploration (global search) and exploitation (local refinement). By integrating random search operators with gradient-informed adjustments, SOA achieves a dynamic balance between broad solution space exploration and precise local optimization and shows several key advantages, such as rapid convergence rate, enhanced escape capability, and robust adaptability, abilities that have led to its successful application in fields of engineering, artificial intelligence (AI), healthcare, etc. In this study, we employed the SOA to optimize hyperparameter combinations in SVR modeling (SOA-SVR models for short). The detailed procedure of using SOA to optimize the three hyperparameters (C, ε, *γ*) of SVR and SVR modeling is shown in Figure 2. The specific steps of SOA-SVR modeling included (1) performing spectral standardization via the standardscaler function in Scikit-learn library within a Python 3.11 environment; (2) screening key features through kernel principal component analysis or mutual information method; (3) searching for an optimal parameter combination (C, ε, *γ*) iteratively by using the SOA algorithm; (4) fully training the optimal parameters and selecting the kernel function (i.e., SVR modeling); (5) conducting a fitness evaluation via 5-fold cross-validation; and (6) conducting model performance evaluation using correlation coefficient (R_CV_, R_P_), root mean square error (RMSE_CV_, RMSE_P_), and relative percent deviation (RPD) based on cross-validation and prediction dataset. The entire computational framework was implemented in Python 3.11 using object-oriented programming, with Scikit-learn’s SVR implementation integrated into the optimization process. The SOA-optimized SVR modeling enabled automated identification of optimal solutions that maximize prediction accuracy while minimizing computational expense.

A random forest (RF) is an ensemble learning algorithm based on decision trees. By constructing multiple classification regression trees (CARTs) and combining Bagging and random feature selection strategies, an RF can enhance the generalization ability and stability of a model [28]. With powerful nonlinear modeling and anti-noise capabilities, RF has become an important tool for solving complex classification and regression problems in NIR analysis. An artificial neural network (ANN) is a computational model that simulates the structure of biological neurons. It analyzes complex data relationships through multi-layer nonlinear transformations and offers significant advantages in NIR analysis. In particular, ANNs can directly process full-band high-dimensional spectral data, avoiding information loss caused by feature selection and greatly improving the analysis efficiency. By optimizing parameters through hybrid genetic algorithms and enhancing the interpretability of a model, ANNs can be used in industrial scenarios involving portable NIR devices, promoting the development of non-destructive testing technology towards intelligence [29].

In this study, SVR, RF, and ANN were applied for modeling by inputting the matrices of reference and NIR spectra, and their performances in predicting SV and FN values were compared in terms of R_CV_, R_P_, RMSE_CV_, and RMSE_P_ and RPD.

### 2.5. Wavelength Selection and Model Simplification

NIR spectra can comprise hundreds or thousands of wavelengths, which exhibit varying degrees of redundancy, noise, and/or analytical irrelevance. This spectral complexity necessitates carrying out wavelength selection as a critical preprocessing step to achieve threefold optimization, including enhancing performance through elimination of non-contributory variables, ensuring computational efficiency via dimensionality reduction, and improving interpretability by focusing on physically meaningful spectral regions. Specifically, the identification of the most informative wavelengths (MIWs) demonstrating the largest contributions with target analytes can enable the construction of simple and robust calibration models while maintaining spectroscopic fidelity [30].

Four effective feature selection methodologies and tools, namely, principal component analysis (PCA), successive projections algorithm (SPA), improved whale optimization algorithm (iWOA), and recursive feature elimination (RFE), were employed both individually and synergistically to identify the MIWs. PCA projects the original high-dimensional data into the low-dimensional orthogonal principal component space through orthogonal transformation, eliminating redundant information and retaining the maximum variance feature. Effective spectral-data noise reduction is achieved by discarding the low-variance principal components (usually created via instrument noise or environmental interference) [31]. SPA operates as a forward variable selection technique that systematically constructs minimally collinear-wavelength subsets through iterative projection operations in vector space. By maximizing spectral information content while minimizing redundancy, this method effectively addresses the “curse of dimensionality” prevalent in NIR spectral datasets, which makes SPA particularly suitable for high-dimensional spectral data processing [32]. WOA, originally proposed by Mirjalili and Lewis, mimics humpback whales’ unique hunting strategies through three mathematically modeled behaviors: prey encircling using shrinking circles, spiral bubble-net attacking via logarithmic spiral motion, and random prey searching through random displacement [33]. This biologically inspired metaheuristic algorithm exhibits balanced global exploration and local exploitation capabilities in combinatorial optimization problems. Building upon the conventional WOA framework, Wang and Gao developed an enhanced version, namely, iWOA, incorporating three critical improvements: chaotic mapping initialization to ensure optimal population diversity, nonlinear time-varying parameters regulated by a Sigmoid transfer function for dynamic adaptation, and greedy selection mechanisms to accelerate convergence [34]. These modifications collectively enhance computational efficiency compared to the original WOA while maintaining high solution accuracy. RFE is a supervised feature selection methodology in which feature subsets are iteratively optimized through the recursive elimination of the least-contributory features. This backward selection process progressively removes features with minimal predictive importance while retaining those exhibiting high discriminative power. By systematically eliminating redundant or noisy features through successive iterations, RFE can effectively enhance a model’s generalization capability and computational efficiency while maintaining essential feature interpretability [35].

After MIW selection, the MIWs were input as predictor variables to simplify the initial SOA-SVR models. The MIW selection procedure was implemented via Scikit-learn’s feature_selection module within a Python 3.11.5 environment.

### 2.6. Independent Validation

NIR calibration models fundamentally rely on training datasets containing chemically characterized reference samples to establish spectral-property correlations. However, complete dependence on training set performance metrics creates model validation blind spots; as such, in-sample evaluations cannot detect overfitting manifestations [36], requiring rigorous out-of-sample testing through completely independent validation sets to achieve objective quantification. In our study, 50 flour samples were randomly collected as input variables to validate the simplified SOA-SVR model, and its performance was evaluated using the parameters mentioned in Section 2.4.

### 2.7. Two-Sample F-Test and t-Test

In chemometric model validation, a two-sample F-test and Student’s *t*-test form the basis of critical hypothesis testing for evaluating spectral predictive precision. The F-test rigorously quantifies variance homogeneity between predicted and reference value distributions, with a rejection of the null hypothesis indicating significant error dispersion discrepancies. The two-sample *t*-test examines systematic prediction bias through mean value comparison, requiring satisfaction of normality and independence assumptions [37]. The two tests were conducted to validate the model’s reliability and carried out in Microsoft Excel 2019.

## 3. Results and Discussions

### 3.1. NIR Features of Wheat Flour

**Figure 3:**
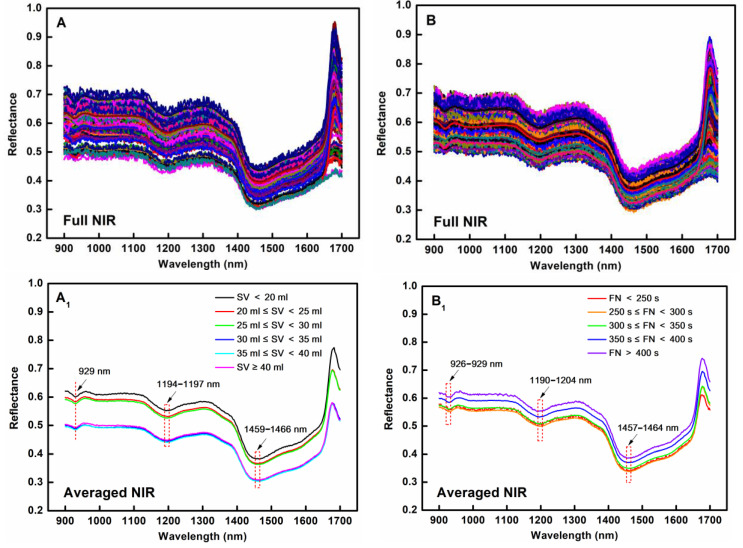

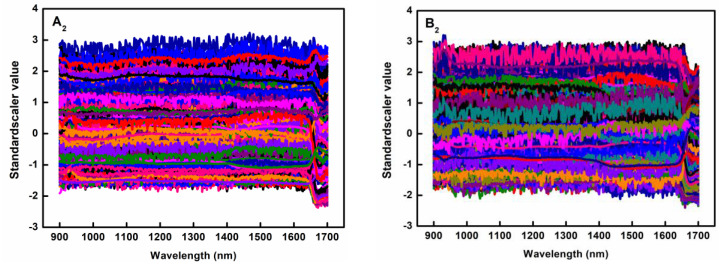
Raw, mean, and standardscaler values of NIR spectral characteristics of wheat flour samples for SV (**A**,**A_1_**,**A_2_**) and FN (**B**,**B_1_**,**B_2_**), respectively.

The raw average (Figure 3A,B) and preprocessed NIR spectra (Figure 3A_2_,B_2_) of the wheat flour samples, with wavelengths ranging from 900 nm to 1700 nm, for SV and FN prediction were profiled, and their signatures are shown in Figure 3. It can be seen that all the spectral curves demonstrated characteristic absorption patterns at different concentrations (Figure 3A_1_,B_1_), with three obvious peaks at around 930 nm, 1200 nm, and 1460 nm. These absorption peaks were mainly caused by the stretching vibrations of hydrogen-containing bonds, X-H (X = C, N, O), in water molecules and organic compounds. Specifically, the peak at around 930 nm resulted from the third overtone of O-H stretching and combination bands corresponding to C-H stretching vibrations in carbohydrates [38]. The peak at around 1200 nm arose from the second overtone of C-H stretching coupled with O-H-bending overtone transitions. The peak at around 1460 nm was attributed to the first overtone of O-H stretching in water molecules [39]. It was also found that the positions of these peaks varied slightly, which may have been due to the concentration variations of the chemical components in the wheat flour samples.

While distinct spectral signatures specifically corresponding to SV and FN were not found in the 900–1700 nm range, we were able to employ AI-driven machine learning techniques, such as the SOA-SVR algorithm, to mine valuable NIR information to quantify the SV and FN of wheat flour, thus allowing the evaluation of wheat flour processing applicability.

### 3.2. Statistical Results Regarding SV and FN

**Figure 4:**
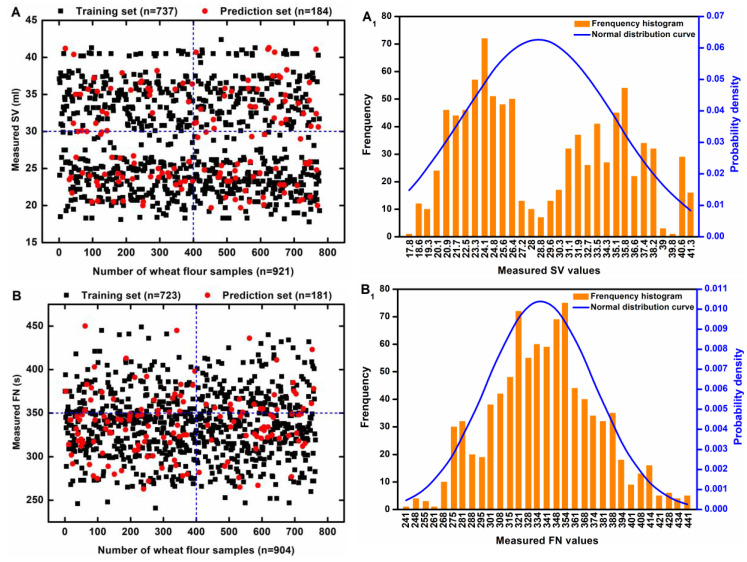
Scatter and normal distributions of measured SV (**A**,**A_1_**), and FN (**B**,**B_1_**) values.

After performing statistical analysis, we found that all the measured values for the two indexes followed a normal distribution; they are illustrated in Figure 4. Figure 4A shows a scatter distribution of the measured SV values used for training (black spots) and prediction (red spots), while Figure 4B displays the FN values. As for Figure 4A_1_, the histogram (with the *y* axis on the left) presented in A_1_ indicates the frequency numbers of the segmented interval data from the measured SV values. The *y* axis on the right shows the normal distribution of the measured SV values. Similarly, the results of the FN analysis are exhibited in Figure 4B_1_.

### 3.3. SOA-SVR Modeling for Quantifying SV and FN Using Full-Band Spectra

The training dataset, constructed as a matrix of samples × spectra, was input and subjected to processing via SOA-SVR, RF, and ANN procedures. Details on the performance of the SOA-SVR model, RF model, and ANN model in predicting SV and FN values are shown in Table 2. As can be seen, the three machine learning models developed using the full-range NIR spectra exhibited different predictive capabilities for both target parameters (SV and FN), with the SOA-SVR model performing better in prediction. The R_P_ exceeded 0.94, and there was a small RMSE_P_ value. Furthermore, the two SOA-SVR models achieved RPD values exceeding 3.0, confirming their suitability for industrial quality control applications [40]. This performance consistency was maintained across 5-fold iterative cross-validation trials, demonstrating the robustness of the method against spectral variance. By contrast, the RF and ANN models showed weaker performance, with smaller R_P_ values and larger RMSE_P_ values. This indicated that SOA-SVR was more suitable for mining spectral information related to the two parameters analyzed.

In recent studies, partial least square (PLS) modeling was performed to predict SV values with a determination coefficient (R_P_^2^) of 0.9055 and an RMSE_P_ of 0.06 (%, *w*/*w*) using 926–1633 nm wavelengths [41] and an R_P_ of 0.896 and an RMSE_P_ of 1.541 (mL) using 1400–2050 nm [42]. These results are inferior to those yielded by our SOA-SVR-driven approach utilizing 900–1700 nm spectra, and this may be due to the different modeling algorithm and wavelength range involved in our spectral analysis. Although Lancelot et al. achieved better performance in FN prediction via PLS modeling based on 1000–2500 nm range data, no prediction datasets were applied for model validation [43], which might have led to a prediction model with poor robustness and reliability. In general, the performance of the final predictive model is affected by multiple factors, such as the modeling algorithm used, NIR spectral data, model validation, etc.

As the best performance was exhibited by the SOA-SVR model, we selected the following wavelengths and made the following model simplifications based on the SOA-SVR model.

### 3.4. Selection of MIWs Through PCA, SPA, iWOA, and RFE

To optimize the computational efficiency of the full-wavelength-based SOA-SVR models while preserving predictive accuracy for SV and FN, multiple algorithms, including CA, SPA, iWOA, and RFE, were employed alone or in combination to identify MIWs. The detailed results are shown in Table 3. For SV, 20 different MIWs were selected via PCA alone or iWOA/SPA, with most of the MIWs concentrated within the range of 1400–1700 nm. This indicated that the 1400–1700 nm range had the most effective spectral information related to the prediction of SV.

For FN, 30 individual MIWs were selected via PCA alone or RFE/iWOA, with almost all the MIWs concentrated within the ranges of 900–1100 nm and 1400–1700 nm. This indicated that the spectra within the two ranges carried the information most relevant to the prediction of FN.

In a word, more than 90% of the wavelengths from the original 360 wavelengths were excluded after MIW selection.

### 3.5. SOA-SVR Modeling for Quantifying SV and FN Using MIWs

By inputting a new data matrix of samples × MIWs (SV: 737 samples × 20 MIWs; FN: 723 samples × 30 MIWs) to execute the SOA-SVR algorithm, we constructed a simplified SOA-SVR model. The model’s predictive ability was evaluated in terms of R_CV_, R_P_, RMSE_CV_, RMSE_P_, and RPD, and the details on the performance for each index (SV and FN) are shown in Table 2. As indicated, the MIW-based SOA-SVR models (SV: iWOA/SPA-SOA-SVR model; FN: RFE/iWOA-SOA-SVR model) performed better and demonstrated similarly good predictive capacity, comparable to that of the full-wavelength-based SOA-SVR model, for either SV or FN prediction. Notably, a reduction in spectral dimensions of more than 90% did not significantly affect model accuracy, confirming the effectiveness of MIW selection in eliminating redundant spectral information while preserving critical SV- and FN-related features.

### 3.6. Simplified SOA-SVR Model Validation Using Independent Samples

**Figure 5:**
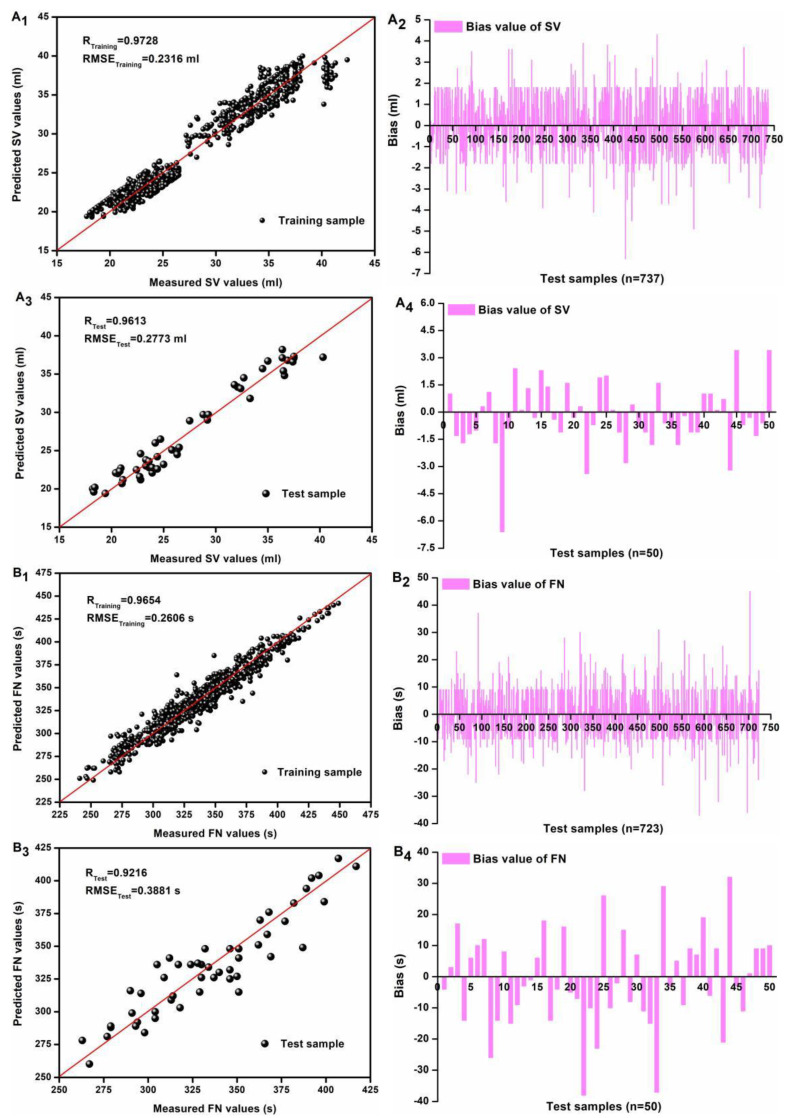
Training and test accuracy (**A_1_**,**A_3_**) and errors (**A_2_**,**A_4_**) regarding iWOA/SPA-SOA-SVR model for SV (mL) and those (accuracy, (**B_1_**,**B_3_**); errors, (**B_2_**,**B_4_**)) regarding iWOA/SPA-SOA-SVR model for FN (s), respectively.

To rigorously assess the robustness and reliability of the two simplified SOA-SVR models, an independent test set consisting of 50 wheat flour samples per index was subjected to model evaluation. The training and validation protocol consisted of applying the iWOA/SPA-SOA-SVR model for SV prediction while employing the RFE/iWOA-SOA-SVR model for FN determination. The training and validation results are shown in Figure 5. The SV and FN values of the test samples were all well predicted (Figure 5A_1_,A_3_,B_1_,B_3_), with over 95% of the bias being less than 10% in training (Figure 5A_2_) and 90% of the bias being less than 10% in testing (Figure 5A_4_) for SV and more than 99% of bias being less than 10% in training (Figure 5B_2_) and 90% of bias being less than 10% in testing (Figure 5B_4_) for FN.

### 3.7. Two-Sample Test

To validate the two simplified SOA-SVR models statistically, an F-test and a *t*-test were employed, and the results are exhibited in Table 4. The F-test revealed non-significant variance discrepancies between the measured and predicted values for the two quality parameters, with an F value < F ‘one-tailed critical value’ (SV: 1.0791 < 1.6073; FN: 1.1134 ≤ 1.6073). This statistical consistency was further confirmed by *t*-test results demonstrating *t* Stat < *t* ‘two-tailed critical value’ across all comparisons (SV: 0.2020 < 1.9845; FN: 0.1027 < 1.9845).

These tests demonstrated the technical feasibility of employing simplified SOA-SVR models for the rapid quantification of SV and FN in wheat flour through portable NIR devices.

## 4. Conclusions

This study investigated the application of an SOA-SVR-driven portable miniaturized NIR spectrometer for the rapid quantitation of the SV and FN of wheat flour. The analytical framework incorporated three critical components: (1) spectra acquisition and feature extraction within the 900–1700 nm range, (2) SOA-SVR modeling to establish quantitative correlations between spectral data and reference measurements, and (3) systematic model optimization through wavelength selection. Primary modeling using an SOA-SVR algorithm with full-range spectra demonstrated promising predictive capabilities, achieving an R_P_ and RMSE_P_ of 0.9752 and 0.2138 mL for SV and 0.9497 and 0.2930 s for FN. Subsequently, iWOA combined with SPA was employed to identify 20 MIWs for SV quantification, while RFE in tandem with iWOA identified 30 MIWs for FN prediction. Based on the MIWs, the simplified SOA-SVR models (iWOA/SPA-SOA-SVR model for SV, RFE/iWOA-SOA-SVR model for FN) maintained a prediction accuracy comparable to that of all the full-range spectra SOA-SVR models (SV: R_P_ = 0.9605, RMSE_P_ = 0.2687 mL; FN: R_P_ = 0.9224, RMSE_P_ = 0.3615 s). External validation using independent test samples combined with two-sample tests (F-test, *t*-test) confirmed the model’s robustness, showing no statistically significant differences between the predicted and reference values (SV and FN). This research advances portable NIR applications in cereal quality control by establishing the first validated quantification method for SV and FN determination in relation to wheat flour and demonstrating the feasibility of combining a miniaturized spectroscope with machine learning to conduct rapid, on-site flour quality assessment.

While NIR spectroscopy has become powerful in regard to food quality monitoring, the existing literature reveals several limitations in the context of wheat flour processing, such as a predominant reliance on benchtop NIR systems rather than field-deployable micro-spectrometers, a lack of validated protocols for the real-time prediction of dough functionality markers (e.g., SV and FN), and the absence of spectral databases correlating miniaturized device outputs with industrial processing parameters. This study makes two novel contributions to the quality analysis of wheat flour: first, it provides the first technical framework integrating a handheld micro NIR spectrometer with SOA-SVR algorithms for online SV and FN quantification, and, second, it provides a methodological innovation in spectral data compression through AI algorithms to enable reductions in most of a dataset without incurring predictive precision loss.

Nevertheless, several critical challenges requiring resolution should be addressed to enable practical implementation of miniaturized NIR systems, such as spectral variability across wheat cultivars, the optimal selection of minimally redundant wavelength combinations, and the development of adaptive algorithms with enhanced cross-variety robustness. Future progress is expected to emerge from synergistic advancements in three key aspects: computational power (enabling real-time multivariate analysis), model migration (strengthening model robustness and generalization ability), and cost-effective manufacturing (reducing per-unit expenditure). Such technological evolution is likely to catalyze the transition of AI-enhanced portable NIR systems from laboratory prototypes to dual-mode industrial solutions capable of both online process monitoring and point-of-sale quality verification.

## Figures and Tables

**Table 1:** Performance of NIR combined with different machine learning algorithms for quality evaluation of wheat flour.

Index	NIR Range	Modeling Methods	Accuracy	Year	Literature
Protein Moisture	400–2500 nm	LGAKNet	R^2^_P_ = 0.9653–0.9683 RMSEP = 0.2886–0.3016 g/100 g RPD = 5.1046–5.8981	2024	[19]
Azodicarbonamide Talcum powder Gypsum powder	11,542–3946 cm^−1^	CNN-SVM	R^2^_P_ = 0.9226–0.9786 RMSEP = 0.0024–1.6506% RPD = 3.5852–6.8368	2024	[20]
Azodicarbonamide	400–2500 nm	GBDT	R^2^_P_ = 0.9778 RMSEP = 0.8905% RPD = 6.8099	2022	[21]
Azodicarbonamide	400–2500 nm	BRR	R^2^_P_ = 0.9802 RMSEP = 0.8914% RPD = 6.9263	2022	[22]
Fatty acid value	899.22–1724 nm	ELM	R^2^_P_ ≥ 0.96 RMSEP ≤ 1.0677 mg KOH/100 g	2020	[23]

Note: LGAKNet, Lightweight Ghost Attention Kolmogorov–Arnold Network; R^2^_P_, determination coefficient of prediction; RMSEP, root mean square error of prediction; RPD, relative prediction deviation; CNN-SVM, convolutional neural network support vector machine; GBDT, gradient boosting decision tree; BRR, Bayesian ridge regression; ELM, extreme learning machine; RF, random forest.

**Table 2:** Performance of SOA-SVR, RF, and ANN models in predicting SV (mL) and FN (s) using the full-range spectra based on training/prediction size of 80%/20% and MIWs, respectively.

Index	Training/Prediction Dataset	Number of Wavelengths	Model	Optimal Value of SVR Hyperparameters			Cross-Validation Performance		Predictive Performance			Training Time (s)
				*C*	*ε*	*γ*	R_CV_	RMSE_CV_	R_P_	RMSE_P_	RPD	
SV	737/184 (80%/20%)	360	SOA-SVR	27.5879	0.1117	0.0032	0.9654	0.2313	0.9752	0.2138	4.5144	56.6724
			RF	—	—	—	0.9080	0.2999	0.9149	0.2856	3.4295	36.2069
			ANN	—	—	—	0.9168	0.2863	0.9302	0.2586	3.7871	1.2409
		20	PCA-SOA-SVR	26.0758	0.3452	0.5789	0.9038	0.3026	0.9088	0.2873	3.3124	24.2079
			iWOA/SPA-SOA-SVR	7.7651	0.2816	0.2451	0.9586	0.2738	0.9605	0.2687	3.5930	53.8704
FN	723/181 (80%/20%)	360	SOA-SVR	314.5539	0.1862	0.0010	0.9333	0.2909	0.9497	0.2930	3.1940	72.1404
			RF	—	—	—	0.7153	0.5296	0.7364	0.5325	1.9479	36.5129
			ANN	—	—	—	0.8680	0.3605	0.8557	0.3686	2.6332	1.2204
		30	PCA-SOA-SVR	887.69	0.7946	0.001	0.7396	0.4912	0.7471	0.4795	1.9888	23.1074
			RFE/iWOA-SOA-SVR	23.8939	0.2424	0.0485	0.9218	0.3643	0.9224	0.3615	2.5894	50.1561

**Table 3:** The MIWs selected via PCA, SPA, iWOA, and RFE for SV and FN prediction.

Index	Method	MIWs	Wavelength Reduction
SV	PCA	908, 1107, 1199, 1416, 1520, 1528, 1545, 1547, 1575, 1611, 1613, 1627, 1629, 1633, 1645, 1655, 1657, 1660, 1662, and 1664 nm	94.44%
	iWOA/SPA	905, 921, 944, 1014, 1046, 1097, 1277, 1347, 1365, 1427, 1451, 1506, 1520, 1545, 1556, 1607, 1619, 1645, 1670, and 1683 nm	94.44%
FN	PCA	911, 931, 946, 964, 972, 982, 987, 1006, 1009, 1021, 1034, 1040, 1056, 1063, 1497, 1510, 1520, 1554, 1571, 1581, 1593, 1595, 1603, 1605, 1611, 1615, 1623, 1627, 1635, and 1641 nm	91.67%
	RFE/iWOA	900, 905, 913, 926, 1051, 1185, 1356, 1393, 1400, 1410, 1423,1455, 1459, 1479, 1497, 1506, 1517, 1561, 1603, 1613, 1625, 1629, 1664, 1680, 1682, 1683, 1687, 1689, 1693, and 1694 nm	91.67%

**Table 4:** Two-sample test results for the measured and predicted values of SV for the iWOA/SPA-SOA-SVR model and FN for the RFE/iWOA-SOA-SVR model.

Index	Model	Test	Item	Measured Value	Predicted Value
SV	iWOA/SPA-SOA-SVR	F-test	Average	29.0	28.7
			Variance	42.1	39.0
			Observed value	50	50
			d*f*	49	49
			*F*	1.0791	
			*P* (*F* ≤ *f*) one-tailed	0.3955	
			F ‘one-tailed critical value’	1.6073	
		*t*-test	Average	29.0	28.7
			Variance	42.1	39.0
			Observed value	50	50
			Merger of variance	40.6	
			Assumed mean difference	0	
			d*f*	98	
			*t* Stat	0.2020	
			*P* (T ≤ t) one-tailed	0.4202	
			*t* ‘one-tailed critical value’	1.6606	
			*P* (T ≤ t) two-tailed	0.8404	
			*t* ‘two-tailed critical value’	1.9845	
FN	RFE/iWOA-SOA-SVR	F-test	Average	334	334
			Variance	1518	1363
			Observed value	50	50
			d*f*	49	49
			*F*	1.1134	
			*P* (*F* ≤ *f*) one-tailed	0.3542	
			F ‘one-tailed critical value’	1.6073	
		*t*-test	Average	334	334
			Variance	1518	1363
			Observed value	50	50
			Merger of variance	1440	
			Assumed mean difference	0	
			d*f*	98	
			*t* Stat	0.1027	
			*P* (T ≤ t) one-tailed	0.4592	
			*t* ‘one-tailed critical value’	1.6606	
			*P* (T ≤ t) two-tailed	0.9184	
			*t* ‘two-tailed critical value’	1.9845	

## Data Availability

The original contributions presented in this study are included in the article. Further inquiries can be directed to the corresponding authors.
